# An Epidemiological Survey of Fluid Resuscitation Practices for Adult Burns Patients in the United Kingdom

**DOI:** 10.3390/ebj6030040

**Published:** 2025-07-09

**Authors:** Ascanio Tridente, Joanne Lloyd, Pete Saggers, Nicole Lee, Brendan Sloan, Kathryn Puxty, Kayvan Shokrollahi, Nina C. Dempsey

**Affiliations:** 1 Intensive Care Unit, Whiston Hospital, Mersey & West Lancashire NHS Trust, Prescot L35 5DR, UK; 2 St. Andrew’s Burns Centre, Broomfield Hospital, Chelmsford CM1 7ET, UK; 3 London & Southeast Burns Network, Chelsea and Westminster Hospitals, 369 Fulham Road, London SW10 9NH, UK; 4 Yorkshire Burn Centre, Pinderfields Hospital, Wakefield WF1 4DG, UK; 5 Intensive Care Unit, Glasgow Royal Infirmary, Glasgow G4 0SF, UK; 6 Mersey Regional Burns Unit, Mersey & West Lancashire NHS Trust, Prescot L35 5DR, UK; 7 Department of Life Sciences, Manchester Metropolitan University, Manchester M15 6BX, UK

**Keywords:** burns, resuscitation, fluid, mortality, outcome, critical care

## Abstract

Fluid management is a critical component in the treatment of patients suffering with major burns. Clinicians must carefully balance judicious resuscitation with the risks of over- or under-resuscitation. We aimed to identify factors associated with survival in burns patients and determine the importance of resuscitation practices. Patients requiring admission to Burns Services in the United Kingdom between 1 April 2022 and 31 March 2023 were included in the National Burns Audit project on fluid resuscitation practices, to evaluate factors associated with survival and Critical Care Length of Stay (CCLoS). A total of 198 patients were included in the analyses, with median age of 51 years (interquartile range, (IQR) 35–62 years), median Total Burn Surface Area (TBSA%) of 27.5% (IQR 20–40%), and median Baux score 82.5 (IQR 66–105). The following were found to be significant for survival: younger age, smaller TBSA%, lower Baux score and independence from renal replacement therapy. Neither the mechanism of burns nor the fluid resuscitation volumes appeared to influence survival. Although interventions such as tracheostomy or the number of surgical procedures did not appear to affect survival, fluid replacement of more than 6 mL/kg/%TBSA independently predicted longer CCLoS. Volume of fluid resuscitation, within the limits examined in this cohort, did not impact likelihood of survival.

## 1. Introduction

Fluid management is a critical component in the treatment of patients suffering with major burns [1]. It has long been reported that fluid resuscitation influences survival rates, although the available literature yields inconsistent and sometimes conflicting results [2,3,4]. Despite the existence of long-established guidelines, controversies persist regarding the optimal fluid strategies, types and volumes to be administered within the first 24 h following burns injury [2,5,6,7]. The fundamental challenge to clinicians is represented by the need to carefully balance adequate and judicious resuscitation with the potential risks associated with over- or under-resuscitation.

The Brooke formula was first developed in 1953 by Reiss et al. [8] and was based on the patient’s body weight in kilograms and percentage body surface burned (%TBSA) to estimate the amount of fluid resuscitation a patient requires immediately following a burn injury. This stated that 1.5 mL/kg/%TBSA of Lactated Ringer’s solution plus 0.5 mL/kg/%TBSA of colloid and 2 L of 5% dextrose in water should be administered within the first 24 h post-injury. This was later modified to 2 mL/kg/%TBSA of Lactated Ringer’s without colloid (reserved for after 24 h post injury) and termed the modified Brooke formula [9]. The Parkland formula was introduced in 1968 by Baxter and Shires and has remained a foundational guideline for fluid resuscitation in burn patients [10]. It recommends administering 4 mL/kg/%TBSA of lactated Ringer’s solution, with half given in the first eight hours and the remainder over the next 16 h [11]. However, despite widespread adoption, there is growing concern that patients may receive excessive fluid volumes, and that this itself may result in poorer outcomes [2,3]. The potential for over-resuscitation is due to a combination of factors, including variance in assessment of %TBSA, individual patient needs, and clinical judgement. The American Burn Association guideline recommends 2–4 mL/kg/%TBSA burn [12], and the American Burn Association Clinical Practice Guidelines on Burn Shock Resuscitation recommends a strategy of fluid resuscitation starting at 2 mL/kg/%TBSA [13], although such recommendation is made on the basis of small studies, and in the absence of strong prospectively gathered evidence [14,15].

One of the primary concerns related to over-resuscitation is “fluid creep”, where excessive fluid administration is complicated by pulmonary oedema, increased intra-abdominal pressure and compartment syndromes, which can be associated with overzealous initial resuscitation practices [16]. Patients with inhalation injury add a further layer of complexity. Inhalation injury in the context of severe cutaneous burns is associated with higher fluid requirements, but excessive fluid replacement may result in airway oedema and may potentially increase the risk of acute respiratory distress syndrome (ARDS) [17]. Depth of burn injury may also influence fluid loss, with deeper injuries generally associated with more severe fluid leakage and more extensive inflammatory response [18,19].

A goal-directed approach to fluid therapy, focusing on specific targets such as urine output and hemodynamic parameters, has more recently been considered. Goal-directed methods endeavour to tailor resuscitation strategies based on individual patient physiological responses, rather than adhering to formulaic guidance, but the scientific community has not reached consensus on their utilisation [20,21].

The relationship between fluid administration and burn outcomes is undoubtedly complex. Burn-related deaths are often due to factors such as sepsis, multi-organ failure, and inhalation injury, which may not be directly related to initial fluid resuscitation volumes. There are also many patient factors such as age and specific comorbidities that may be more important in determining outcome, irrespective of adequate fluid resuscitation, and some patients may require larger volumes of fluid to maintain adequate organ perfusion without experiencing negative consequences [22].

This first and wide-ranging epidemiological survey of burns patients in the United Kingdom is the only comprehensive analysis of a full UK national cohort of burn resuscitation data ever to be published and allowed us to elucidate the recent and real-world patterns of initial fluid resuscitation practice (within the first 24 h) and to understand associated patient outcomes.

## 2. Materials and Methods

### 2.1. Study Design, Setting, and Inclusion Criteria

This survey examined data from the prospectively gathered Burns National Audit cohort of patients, requiring admission for burns management and fluid resuscitation, to burns critical care services in England and Wales (including 14 adult centres and units), and from Northern Ireland (Belfast), between 1 April 2022 and 31 March 2023. Inclusion criteria were age 16 years or older, admission to a burns service for fluid resuscitation due to a burns total body surface area (TBSA) ≥ 15%. The 15 burns services that contributed to data collections were: St Andrew’s, Broomfield Hospital (Chelmsford), Chelsea and Westminster Hospital (London), Stoke Mandeville Hospital (Aylesbury), Queen Victoria Hospital (East Grinstead), Queen Elizabeth Hospital (Birmingham), Nottingham University Hospital (Nottingham), Wythenshawe Hospital (Manchester), Royal Victoria Infirmary (Newcastle), Pinderfields Hospital (Wakefield), Whiston Hospital (Prescot), Northern General Hospital (Sheffield), Southmead Hospital (Bristol), Swansea Bay University Health Board (Swansea), Salisbury District Hospital (Salisbury) and Royal Victoria Hospital (Belfast).

### 2.2. Data Collection

Data including date of admission, burns service, patient demographics (age and sex), weight on admission, and TBSA were recorded on admission, as well as mechanism of burn, volume used in the calculations of total resuscitation fluid requirements for the Parkland formula calculations (whether 2, 3 or 4 mL/kg/%TBSA), actual total fluid input for the first 24 h (calculated as mL/kg of body weight per % of burn surface (mL/kg/%TBSA)), total urine output in the first 24 h (mL/kg/h), performance of a tracheostomy, number of surgical procedures, need for renal replacement therapy (RRT), length of burns critical care stay (CCLoS) and hospital stay (HLoS), and survival at discharge from hospital (hospital survival). We examined hospital survival as primary outcome, and CCLoS as secondary outcome.

### 2.3. Patient Population

Data for 217 adult patients were collected in total. Following data quality checks, data from 19 patients (including 5 duplicates) were excluded from further analysis:

- Ten patients had burns below the threshold for fluid resuscitation (15% TBSA).
- Five patients had duplicate records: they had been transferred from a Burns service to another for further management, hence were present in the records from both centres involved, and therefore appeared twice in the database (two patients were transferred from Stoke Mandeville to St. Andrew’s, and three from Bristol’s to Swansea’s services). The data for these patients are included only once, as collected more comprehensively by the destination hospitals.
- Four patients treated in burns services had suffered skin loss due to other medical diagnoses but had not suffered burns.

Of the remaining 198 patients, 5 had been treated conservatively, with palliative care, immediately or shortly after presentation, and were considered separately in the relevant analyses: we defined these 5 patients as the “early palliative care” group. This subset of palliative patients was indicated as such in the database by the services, with various comments, such as “non survivable injury, EOL care” (where EOL indicates End of Life), “palliated on arrival to ED” or “Comfort care” (SIC). Sensitivity analyses were performed (with and without the 5 early palliative care patients) to ensure that their inclusion/exclusion did not significantly alter results and/or did not introduce biases in the analyses.

### 2.4. Statistical Analyses

All statistical analyses were performed using STATA 18.5 (StataCorp, Lakeway Drive, College Station, Texas 77845, USA). Data are presented as mean values (and standard deviation, SD), or as medians (and interquartile range, IQR), as appropriate. To explore factors associated with mortality, univariable and multivariable logistic regression analyses were performed. Univariable association filtering was used to select variables to feed into the multivariable model, as commonly performed purposeful selection methodology [23,24]. Statistical interaction was examined using statistical interaction terms and Likelihood ratio testing. To evaluate factors associated with length of stay, univariate and multivariate linear regression analyses were employed. Again, univariate association filtering was utilised to select variables to feed into the multivariate models. A *p*-value cut off 0.05 was used for statistical significance. We assessed correlation between variables using the Pearson correlation coefficient with pairwise comparisons.

## 3. Results

### 3.1. Patient Baseline Characteristics and Fluid Resuscitation

A total of 198 patients, admitted to burns services in England and Wales (including 14 adult centres and units), and from Northern Ireland (Belfast), between 1 April 2022 and 31 March 2023, were included in the analyses. Patient characteristics are described in Table 1. The median age was 51 years (interquartile range, IQR 35–62 years) with a male preponderance, 128 patients being male (64.6% of the cohort).

The patients in the cohort had suffered burns of various origins, with the majority (104, 52.5%) being due to flame, 20 (10.1%) classified as “scalds”, a large group (42, 21.2%) of unclear/non-specified origin, followed by other causes, as indicated in the table. Median TBSA was 27.5% (IQR 20–40%), with the smallest burns being 15% (by inclusion criteria) and largest 98%. The median Baux score was 82.5 (IQR 66–105), and the mean Baux score was 84.5 (standard deviation, SD 27). Fluid resuscitation and urine output data are presented in Table 2.

The median volume of fluid resuscitation received in the first 24 h was 3.43 (IQR 2.50–4.49) mL/kg body weight/%TBSA. Ranges of fluid resuscitation volumes are presented in Table 2, which shows that 65 (approximately a third, 32.9%) patients, whose fluid resuscitation was recorded, received over 4 mL/kg/%TBSA, and 5.5% received over 6 mL/kg/%TBSA. On the contrary, 29 [14.6%] of those patients with known fluid resuscitation regimen received less than 2 mL/kg/%. The median urine output was 0.65 (IQR 0.34–1.19) mL/kg/h. The urine output was highly correlated with the fluid resuscitation volume (as measured by volume per kg body weight per percentage burns area), with Pearson correlation coefficient R = 0.2079, *p* = 0.0056, at pairwise comparison (Figure S1, Supplementary Materials). Twenty nine patients who received less than 2 mL/kg/%TBSA had a median recorded urine output of 0.28 (IQR 0–0.57) mL/kg/h; while 149 patients with resuscitation regimens administering 2 mL/kg/%TBSA or higher volumes had a median recorded urine output of 0.71 (IQR 0.46–1.29) mL/kg/h. Table 3 reports patients’ outcomes, including overall survival, mortality rates stratified by fluid resuscitation volumes, and length of critical care and hospital stay.

When “centres” were included as a variable in the analyses for impact on mortality (the primary outcome), for impact on likelihood of performing a tracheostomy or for initiating renal replacement therapy, we found no correlation between the centre’s variable and any of these other outcomes.

### 3.2. Predictors of Hospital Survival

All five patients (2.5%) palliated on admission died and were excluded from the inferential analyses to establish factors predictive of survival. For those not palliated, univariable logistic regression analyses were conducted to determine predictors of hospital survival. At univariable regression analyses, the following were found to be significant: younger age, smaller TBSA%, smaller Baux score and RRT independence. Neither the mechanism of burns, nor the fluid resuscitation volumes, appeared to be associated with survival. Interventions such as tracheostomy, or the number of surgical procedures, also did not appear to be retained as independent predictors of survival, in our cohort. The variables found to be significant at univariable analyses were fed into the multivariable logistic regression. Age (Odds Ratio [OR] = 0.89, 95% Confidence Interval [CI] = 0.86–0.94, *p* < 0.001), TBSA% (OR = 0.89, CI = 0.86–0.94, *p* < 0.001), Baux score (OR = 0.89, CI = 0.86–0.94, *p* < 0.001) and RRT independence (OR = 0.053, CI = 0.003–0.85, *p* < 0.038) were all retained as significant in the multiple logistic regression, as shown in Table 4.

### 3.3. Predictors of Length of Stay in Critical Care

Univariate and multivariate linear regression analyses were conducted to determine predictors of CCLoS. Results of the univariate analyses are presented in Table 5. The need for tracheostomy, the number of surgical procedures, and an actual fluid resuscitation regimen of more than 6 mL/kg/%TBSA were all associated, at univariate analyses, with a prolonged CCLoS. These factors persisted as independent predictors of increased CCLoS at multivariate regression model.

## 4. Discussion

The management of fluid resuscitation in major burns remains a complex and evolving field. This large, multicentre cohort of adult burns patients offered the opportunity to examine real-life fluid resuscitation practices across the UK. It has demonstrated that there is a significant variability in resuscitation practices across the United Kingdom. Increased fluid resuscitation was associated with increased urine output. Data regarding burns depth, coexistence of inhalational injury, trauma, and other factors which may influence fluid resuscitation practices, was not collected as part of this dataset. It is therefore possible that the variable practice detected at national level was, to some extent, in response to the relevant clinical needs of the patients. For example, superficial burns may need very little fluid resuscitation, while patients affected by deeper burns, smoke inhalation, other associated injuries, or other factors, such as delay in resuscitation, or alcohol ingestion, may typically require more.

A quarter of patients (25.97%) did not survive to hospital discharge, and mortality, when stratified across resuscitation ranges, did not vary significantly across groups, with an increase in mortality only seen for volumes of resuscitation above 6 mL/kg/%TBSA (although this did not reach statistical significance). The median CCLoS and HLoS were 9 (IQR 5–19) and 15 (IQR 9–23) days, respectively.

In line with other published work, younger age, smaller TBSA, lower Baux score and RRT independence were identified as being significantly associated with hospital survival at univariate and multivariate analyses [25,26,27,28]. The lack of association between fluid resuscitation in the first 24 h and survival observed in our study has been reported in several similar studies; Dulhunty et al. explored the relationship between administered fluid volume and development of adverse events and mortality in eighty-one patients with ≥15% TBSA [2]. Despite an attempt to adhere to the Parkland formula, fluid management was seen to differ markedly in terms of fluid volume across the cohort. Yet, it was reported that, while higher fluid volume was associated with pneumonia and extremity compartment syndrome, associations with abdominal compartment syndrome, failures of the intestinal, hepatic, renal, cardiovascular and respiratory systems, and most notably, mortality could not be identified. The study did report that TBSA was independently associated with all these adverse events and with mortality. In a more recent study, Rizzo et al. investigated the impact of different initial fluid formulas (2 mL/kg/TBSA, 3 mL/kg/TBSA, 4 mL/kg/TBSA and volumes calculated using the “Rule of Ten”) on adverse events and seven-day mortality in a cohort of 296 patients with ≥20% TBSA [29]. The “Rule of Ten” is a simplified approach to calculating initial fluid resuscitation rates for adult burn patients in emergency settings where quick estimations are necessary [30]. The study reported that there were no significant differences in rates of complications or mortality between the groups, and again, highlighted the importance of the TBSA and Baux score in predicting outcomes.

In contrasting research by Klein et al., a study carried out on 72 patients with ≥20% TBSA reported that increased fluid volume administered led to increased risk of development of pneumonia, bloodstream infections, ARDS, multiorgan failure and death [3]. Interestingly though, the same study reported that resuscitation volumes of 1.5 times or even twice the volumes expected (based on the Parkland formula) were not significantly associated with these complications or with hospital mortality. It is worth noting that, in this latter study, the mean fluid volume received during the first 24 h after injury was 5.2/mL/kg/TBSA, while the mean volume in our cohort was significantly lower (3.44/mL/kg/%TBSA). Mean urine output was 1.1 mL/kg/h in the study by Klein and colleagues, significantly higher than the mean of 0.88 mL/kg/h in our cohort. This may reflect changes in clinical practice since the publication of the Klein et al. study in 2007, where targeted urine output volumes of 0.5 mL/kg/h are now preferred over volumes >1 mL/kg/h.

A recent study of data from the German Burn Registry involving 2235 burn patients with ≥15% TBSA, investigated the impact of relative deviation from Parkland formula (expressed as percent deviation) on in-hospital mortality [31]. After adjusting for sex, age, BMI, TBSA, the presence of a third-degree burn, and inhalation injury, only positive deviations (i.e., greater fluid volumes) from the formula were found to be significantly associated with increased mortality. The study also reported that the negative effect of excessive fluid administration on mortality was less pronounced in patients with higher TBSA, younger patients and patients with inhalation injury or obesity. The study did not report on urine outputs, and so evaluation of the adequacy of resuscitation is difficult.

Chung et al. evaluated the relationship between the estimated fluid volumes calculated, either by the Modified Brooke or the Parkland formulas and the actual volumes received in 52 military personnel with burn injuries ≥20% TBSA [14]. As expected, overall fluid volumes administered were significantly lower in the Brookes group than the Parkland group. Yet interestingly, incidence of abdominal compartment syndrome and death was not significantly different between the two groups, indicating that what is currently perceived as under-resuscitation does not appear to be associated with poorer outcomes.

From our multivariate analysis, we found that increasing number of surgical procedures, requirement for a tracheostomy and an actual fluid resuscitation regimen of more than 6 mL/kg/%TBSA were all associated with a longer critical care stay, independently of other patient factors including burn size. Thresholds for performing tracheostomies and also initiating RRT may differ among practitioners, but when “centre” was included as a variable in our analyses, we found no impact on mortality, likelihood of performing a tracheostomy or for initiating RRT. A study by Lindahl et al. grouped patients into those who received <6 mL/kg/burn% and those who received >6 mL/kg/burn% during the first 24 h [7]. Consistent with our findings, they reported that, fluid resuscitation regimen was not associated with ICU mortality, yet a fluid resuscitation regimen of >6 mL/kg/burn% was independently associated with longer ICU stay. Interestingly, a higher cumulative fluid volume administrated within the first 72 h was associated with both mortality and longer length of ICU stay, suggesting that lower infusion rates at the start of fluid resuscitation that result in smaller cumulative fluid volumes at 72 h may be beneficial.

Unsurprisingly, an increasing number of surgical procedures has previously been identified as being associated with increased ICU length of stay by several studies, as described in our cohort [32,33,34]. Requirement for tracheostomy has also been shown to be associated with longer ICU stay, and in these studies, it has been reported to be linked to the presence of inhalation injury. The present study did not collect data on inhalation injury. However, a study by Ruiz et al. conducted on a small number of burn patients (n = 33) all with inhalation injury, demonstrated that length of ICU stay was significantly longer in those that required tracheostomy compared with those who did not, despite similar TBSAs between the two groups [35]. It is worth noting that in general, the majority of patients with a tracheostomy can only be treated in ICU, and as a result, the stay of these patients may be artificially lengthened for reasons other than the burn injury. In line with the published literature, our study found no association between requirement for tracheostomy and increased mortality [36,37].

This multicentre study examining fluid resuscitation regimens across 14 burn centres and units allowed for an examination of recent initial fluid resuscitation practices across the UK in a large cohort of burn injuries (n = 198) requiring ICU support. The sample size allowed for adequate power when stratifying patients by fluid resuscitation volumes. Although we observed a significant spread of fluid resuscitation practices used across the cohort, the mean volume administered within 24 h (3.44 mL/kg/%TBSA (1.82)) was seemingly lower than described in many other studies focusing on fluid resuscitation protocols, which have frequently reported mean volumes > 5.0 mL/kg/%TBSA and comparably higher mortality rates [2,3]. Notably, we and others did not find any associations between “under-resuscitation” and either survival or increased length of ICU stay. These findings provide weight to the concept of “permissive hypovolemia” in burns resuscitation. Although studies examining the impact of hypotensive resuscitation are limited, such restrictive fluid strategies may help mitigate complications such as compartment syndromes [38].

Several limitations of the study are recognised. There was difficulty in assessing patient specific factors that may influence decisions regarding fluid resuscitation such as presence of inhalation injury, burn depth and comorbidity details. Indeed, inhalation injury leads to a systemic inflammatory response that worsens capillary permeability beyond that occurring following cutaneous burns alone. This results in greater plasma extravasation and intravascular fluid loss, leading to higher fluid requirements. As previously mentioned, the authors had no availability of data regarding the percentage of full-thickness burns, which would clearly influence fluid requirements. Deeper injuries are generally associated with more severe fluid leakage and greater requirement for fluid replacement. The presence of co-existing trauma may, via additional fluid losses and hemodynamic instability, necessitate increased fluid resuscitation requirements beyond those calculated for burns alone. It is worth noting that alcohol use is frequently associated with burn injury [39] and may independently contribute to polyuria and dehydration, leading to increased fluid requirements. Alcohol consumption data were not available for this study. Although we studied the association of fluid resuscitation regimens on mortality, the influence of different approaches to resuscitation on morbidity could not be explored. Importantly, we had no data on the type of fluid utilised, whether crystalloids, colloids or a combination of these, hence we could not cater for these differences in our analyses. We did not consider the timing of resuscitation and its influence on fluid resuscitation needs and other outcomes. Delayed initiation of fluid resuscitation can increase overall fluid requirements and affect patient outcomes. Therefore, carefully timed fluid administration is as important as the total volume given. We have no data around the timing of burns excision, nor the time period between injury and arrival to the burns services, both of which may have an influence on outcomes. The requirement for immediate surgical intervention, such as early excision or debridement, can significantly influence fluid requirements due to associated blood loss and physiological stress. A further consideration is that our study did not include data on the timing or extent of surgical procedures, which may have impacted fluid administration and confounded resuscitation outcomes. Finally, 11 patients (5.6%) received fluid resuscitation of 6 mL/kg/%TBSA or more. We have no data on whether any of these patients developed pulmonary oedema, extremity or abdominal compartment syndrome.

## 5. Conclusions

The present study sought to elucidate the association between resuscitation practices and outcomes in burns. Although fluid management has been reported to influence survival rates, the available literature yields inconsistent results. Our analyses suggest that, when other potential confounding and explanatory variables are considered, younger age, smaller %TBSA, lower Baux score, and RRT independence may be the independent predictors of survival, but not the volume of fluid resuscitation used or the type of burn. Although these factors provide a framework for predicting outcomes in burn patients, the full clinical context should always be considered when making decisions about treatments, treatment limitations and escalation of care. While these studies question the direct link between initial fluid resuscitation and mortality, they do not negate the importance of appropriate fluid management in burn care. Instead, they highlight the need for individualised, carefully titrated fluid therapy that balances the risks of under-resuscitation against those of fluid overload. A new prospective, multi-centre study, evaluating the impact of different fluid resuscitation strategies and interventions on clinical outcomes, carefully and collaboratively designed to collect additional data (including all potential confounding factors, which may not have been available for the current study), would be highly beneficial and allow us to gain further insights in this important area of burns research.

## Figures and Tables

**Table 1:** Demographic and clinical data for all patients included in the study (n = 198).

**Patient demographics and other characteristics**	
Age, (years), median [IQR]	51 [35–62]
Male sex, n [%]	128 [64.6]
Weight on admission (Kg), median [IQR]	84.1 [70–92]
Burn Total Body Surface Area affected (TBSA%), median [IQR]	27.5 [20–40]
Burn TBSA affected (TBSA%), mean [SD]	34.2 (19.7)
Baux score, median [IQR]	82.5 [66–105]
Baux score, mean [SD]	84.5 [27]
**Mechanism of burn injury**	
Flame, n [%]	104 [52.5]
Scald, n [%]	20 [10.1]
Flash, n [%]	16 [8]
Chemical/contact, n [%]	9 [4.6]
Combined Flash and Flame, n [%]	6 [3]
Electrical, n [%]	1 [0.5]
Unknown/Not specified, n [%]	42 [21.2]
**Patient initial management at admission**	
Palliated on admission, n [%]	5 [2.5]
Actively treated, n [%]	193 [97.5]
**Other management strategies employed during admission**	
Requirement for renal replacement therapy (RRT), n [%]	4 [2]
Requirement for tracheostomy, n [%]	51 [25.8]
Number of surgical procedures, median [IQR]	2 [1–4]

IQR, Inter-Quartile Range; SD, Standard Deviation.

**Table 2:** Fluid resuscitation and urine output data.

**Fluid Resuscitation**	
Volume received in the first 24 h (mL/kg/%TBSA *)	
Median, [IQR]	3.43 (2.50–4.49)
Mean, [SD]	3.44 (1.82)
Categorised (in ranges of mL/kg/%TBSA *) (n, [%])	
0–2	29 [14.6]
2–3	37 [18.7]
3–4	47 [23.7]
4–6	54 [27.3]
Over 6	11 [5.6]
Palliated at presentation	5 [2.5]
Not recorded	15 [7.6]
**Urine Output** (mL/kg/h)	
Calculated urine output, median [IQR]	0.65 [0.34–1.19]
Calculated urine output, mean [SD] (all patients)	0.88 [0.86]
Calculated urine output, median [IQR] (patients receiving </= 2 mL/kg/% fluid resus regimen)	0.28 [0–0.57]
Calculated urine output, mean [SD] (patients receiving </= 2 mL/kg/% fluid resus regimen)	0.34 [0.33]
Calculated urine output, median [IQR] (patients receiving > 2 mL/kg/% fluid resus regimen)	0.71 [0.46–1.29]
Calculated urine output, mean [SD] (patients receiving > 2 mL/kg/% fluid resus regimen)	0.99 [0.9]

* IQR, Inter-Quartile Range; SD, Standard Deviation; mL/kg/%burn indicates the volume administered when indexed for kg of bodyweight on admission, per percentage of TBSA affected by burns; mL/kg/h indicates hourly urine output indexed by body weight.

**Table 3:** Patient outcomes.

Outcomes	
Hospital Mortality (overall)—outcome known N = 154	
Survivors, n [%]	114 [74.0]
Non-survivors, n [%]	40 [26.0]
Hospital Mortality (stratified by resuscitation volume)	
Mortality by resuscitation volume (in ranges of mL/kg/%TBSA *)	n^1^/n^2^ [%] *
0–2	6/27 [22.2]
2–3	7/32 [18.7]
3–4	8/37 [21.6]
4–6	10/43 [23.3]
Over 6	3/9 [33.3]
Palliated at presentation, n [%]	5/5 [100]
Length of Critical Care Unit Stay (days), median [IQR]	9 [5–19]
Length of Hospital Stay (days), median [IQR]	15 [9–23]

* mL/kg/%burn indicates the volume administered when indexed for kg of bodyweight on admission, per percentage of TBSA affected by burns.

**Table 4:** Significant predictors of hospital survival at univariate and multivariate logistic regression analysis.

	Univariate Analysis			Multivariate Analysis		
	OR	95% CI	*p*	Odds Ratio	95% CI	*p*
Age	0.94	0.91–0.96	<0.001	0.89	0.86–0.94	<0.001
Male sex	1.23	0.59–2.69	0.597	-	-	-
Weight on admission	1.02	0.99–1.04	0.063	-	-	-
Burns TBSA%	0.94	0.92–0.97	<0.001	0.89	0.86–0.94	<0.001
Baux	0.90	0.87–0.93	<0.001	0.89	0.86–0.94	<0.001
Tracheostomy	0.78	0.33–1.82	0.559			
Renal Replacement Therapy	0.09	0.01–0.94	0.044	0.05	0.00–0.85	0.038
Number of Surgical procedures	1.15	0.98–1.35	0.083	-	-	-
Mechanism of burn						
Chemical/contact	1.00	-	-	-	-	-
Flame	0.30	0.04–2.49	0.263	-	-	-
Flash	1.00	-	-	-	-	-
Scald	0.50	0.48–5.24	0.563	-	-	-
Electrical	1.00	-	-	-	-	-
Combined Flash and Flame	0.63	0.03–12.41	0.758	-	-	-
Unknown/Not specified	1.00	-	-	-	-	-
Fluid Resuscitation (mL/kg/%TBSA *)						
0–2	1.00	-	-	-	-	-
2–3	1.02	0.30–3.51	0.974	-	-	-
3–4	1.03	0.31–3.43	0.954	-	-	-
4–6	0.94	0.30–2.98	0.920	-	-	-
Over 6	0.57	0.11–2.99	0.508	-	-	-

* mL/kg/%burn indicates the volume administered when indexed for kg of bodyweight on admission, per percentage of TBSA affected by burns. OR = Odds Ratio; CI = Confidence Interval; *p* = Probability.

**Table 5:** Significant predictors of Critical Care Length of Stay at univariate and multivariate linear regression analysis.

	Univariate Analysis			Multivariate Analysis		
	Regression Coefficient	95% CI	*p*	Regression Coefficient	95% CI	*p*
Age	−0.00	−0.14–0.14	0.968	-	-	-
Male sex	−3.93	−9.40–1.60	0.161	-	-	-
Weight on admission	−0.04	−0.19–0.10	0.540	-	-	-
Burns TBSA%	0.12	−0.01–0.26	0.074	-	-	-
Baux	0.07	−0.03–0.17	0.177	-	-	-
Tracheostomy	22.50	17.33–27.67	<0.001	19.41	14.67–24.14	<0.001
Renal Replacement Therapy	−0.86	−19.15–17.43	0.926	-	-	-
Number of Surgical procedures	2.96	2.23–3.68	<0.001	2.29	1.63–2.96	<0.001
Mechanism of burn						
Chemical/contact	0.52	−11.80–12.80	0.934	-	-	-
Flame	−7.81	−22.50–6.90	0.297	-	-	-
Flash	−12.96	−27.10–1.24	0.073	-	-	-
Scald	10.44	−26.80–47.72	0.581	-	-	-
Electrical	−5.56	−25.30–14.16	0.579	-	-	-
Combined Flash and Flame	−6.98	−20.20–6.23	0.298	-	-	-
Unknown/Not specified	1	-	-	-	-	-
Fluid Resuscitation (mL/kg/%TBSA *)						
0–2	1	-	-	-	-	-
2–3	−3.55	−12.47–5.38	0.434	-	-	-
3–4	6.81	−1.71–15.33	0.116	-	-	-
4–6	3.20	−5.107–11.46	0.446	-	-	-
Over 6	15.35	2.68–28.02	0.018	10.86	1.27–20.43	0.027

* mL/kg/%burn indicates the volume administered when indexed for kg of bodyweight on admission, per percentage of TBSA affected by burns. CI = Confidence Interval; *p* = Probability.

## Data Availability

Reasonable requests to access the datasets analysed will be adjudicated by the study collaborators, upon application to the corresponding author.

## Supplementary Materials

The following supporting information can be downloaded at: https://www.mdpi.com/article/10.3390/ebj6030040/s1, Figure S1: Pearson correlation analysis of urine output and fluid resuscitation volume.

## Abbreviations

The following abbreviations are used in this manuscript:

ARDS	Acute Respiratory Distress Syndrome
CCLoS	Critical Care Length of Stay
CI	Confidence Interval
ICU	Intensive Care Unit
IQR	Interquartile Range
OR	Odds Ratio
P	Probability
RRT	Renal Replacement Therapy
SD	Standard Deviation
TBSA	Total Burned Surface Area
