# Supplementary material for: An Epidemiological Survey of Fluid Resuscitation Practices for Adult Burns Patients in the United Kingdom
Source: Eur Burn J. 2025 Jul 9;6(3):40. doi: 10.3390/ebj6030040 (PMC12285936; doi:10.3390/ebj6030040)
Supplement: Supplementary file 1 [file ebj-06-00040-s001.zip › ebj-3733116-supplementary.pdf]

An epidemiological survey of fluid resuscitation practices for adult burns patients in the United Kingdom.

Supplementary Materials - Index

Supplementary Results

Figure S1: Pearson correlation analysis of urine output and fluid resuscitation volume. pag. 2

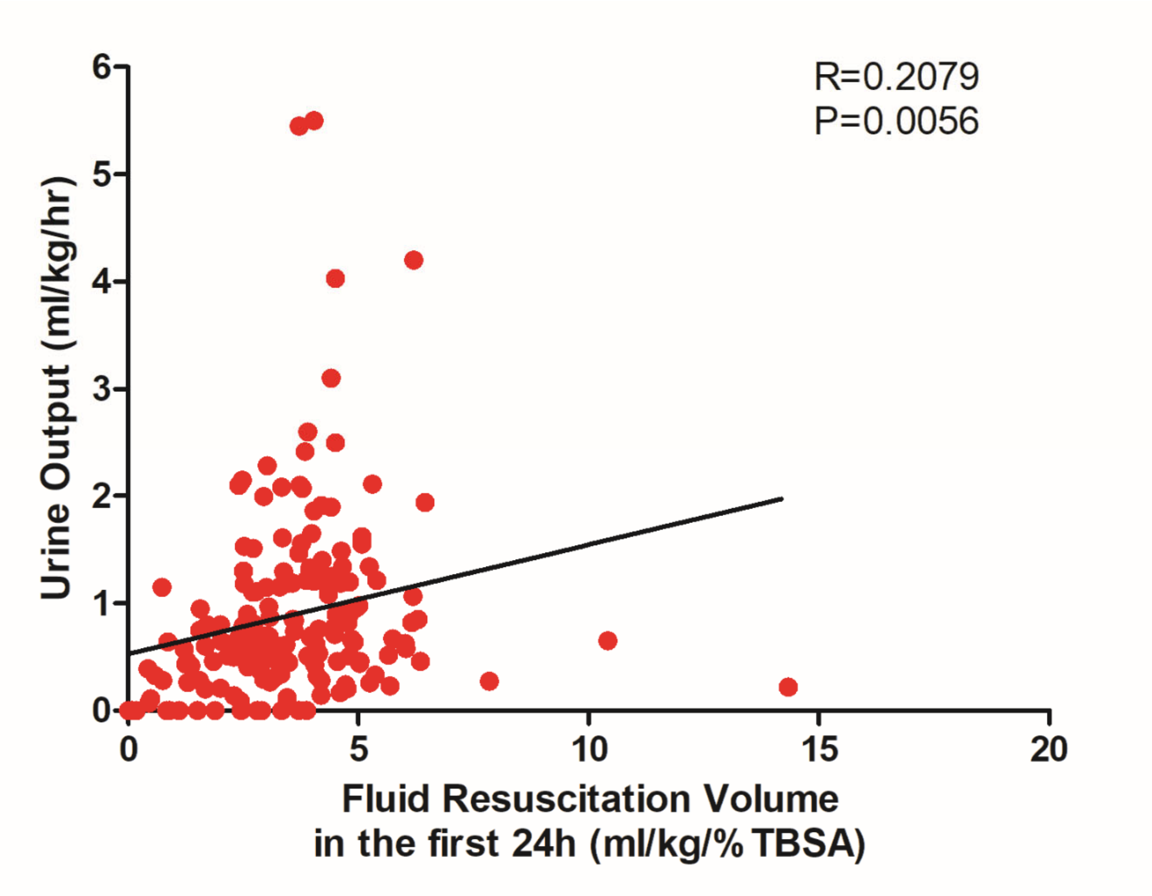

Figure S1: Pearson correlation analysis of urine output and fluid resuscitation volume.
